# Prevalence of dental caries and associated social risk factors among preschool children in Riyadh, Saudi Arabia

**DOI:** 10.12669/pjms.322.9439

**Published:** 2016

**Authors:** Laila A. Al-Meedani, Yousef H. Al-Dlaigan

**Affiliations:** 1 Dr. Laila A. Al-Meedani, BDS. Division of Pediatric Dentistry, Department of Pediatric, Dentistry and Orthodontics Sciences, College of Dentistry, King Saud University, Riyadh, Saudi Arabia; 2 Dr. Yousef H. Al-Dlaigan, BDS, MS, Cert Pedia, PhD. Division of Pediatric Dentistry, Department of Pediatric, Dentistry and Orthodontics Sciences, College of Dentistry, King Saud University, Riyadh, Saudi Arabia

**Keywords:** Children, Caries, Preschool, Parents, Social

## Abstract

**Objectives:**

To determine the prevalence of dental caries, and associated social risk factors among preschool children in Riyadh, Saudi Arabia.

**Methods:**

The study consisted of a random sample of 3 to 5 years- old preschool children who were examined in Riyadh, Saudi Arabia; 388 children (184 boys and 204 girls) were examined from 10 different preschools. Each surface of their teeth was examined for dental caries utilizing modified WHO criteria (WHO, 1997). Data information about age, gender and social factors status were obtained by questionnaires that had been answered by parents.

**Results:**

About 69% of children had dental caries with dmft score of 3.4 (± 3.6) and dmfs of 6.9 (± 9.9). There was no statistically significant difference between boys and girls. Less caries was observed among children whose parents worked and it was statistically significant as well as whose mothers had high or low educational level. Increased number of family members appeared to have a high incidence of dental caries which was also statistically significant. There was no significant difference in dental caries prevalence with birth order.

**Conclusions:**

Dental caries among preschool children in Saudi Arabia was still very common. Improvement of preventive measure at early age should be emphasized by parents and dental health professionals. More attention is required for Non-working parents telling them about the risk of dental caries affecting their children and the awareness of preventive care of dental health.

## INTRODUCTION

In spite of recent improvement in awareness about oral and dental health among public; dental caries remains a significant problem especially in developing countries. Historically; children in developing countries have high caries prevalence; such as China (85%),^1^ India (53%)^2^ and South Africa (49%)^3^ as compared to developed countries such as England (32%)^4^ and Italy (16%).^5^ Since Saudi Arabia is a large, multicultural country; caries prevalence varies in its different regions and cities. However, caries prevalence is high in most regions and cities of Saudi Arabia. A recent study in Jeddah among preschool children found a high caries prevalence of 89%.^6^ A study by Wyne^7^ in Riyadh reported a caries prevalence of 74.8% with a mean dmft score of 6.1 in preschool children.

The environment in which children live and grow up has been reported as an influencing factor on their health behaviors.^8^ Socioeconomic statuses of families have an association with prevalence of dental caries.^9^, ^10^ Families with low socioeconomic status and low educational levels usually have less access to dental services, oral hygiene products; and have poor knowledge about oral hygiene, resulting in greater prevalence and severity of dental caries.^11^ Johnsen et al.^12^ observed that caries-free children had parents with higher educational levels, were aware about prolonged retention of their own dentitions, had smaller families, kept scheduled dental appointments, and low frequency of in-between snacks.

Several researchers have found that mothers with higher educational qualifications have children with better dental health.^13^, ^14^ Eronat and Koparal^15^ reported a lower level of dental caries among Turkish children, whose mothers had high level of education.^15^ Similarly, lower caries prevalence was reported in Jordanian children where the mothers had higher educational levels.^16^ A large family size has also been reported to be associated with higher risk of dental caries in children.^17^, ^18^

In view of the very high caries prevalence in preschool children in Riyadh, it imperative that caries prevalence studies are regularly conducted to determine if there are any changes in the caries prevalence; and to monitor the effectiveness of various caries prevention programs. The caries prevalence studies provide basis for designing caries prevention programs; and treatment needs in the study population. Therefore, the purpose of this study was to determine the prevalence of dental caries, and associated social risk factors among preschool children in Riyadh, Saudi Arabia.

## METHODS

The study utilized stratified random sampling to obtain a fair estimation of caries prevalence in each stratum with minimal sample random error. There are 398 kindergartens/preschools with a total of 23,300 children in Riyadh. The information about kindergartens was obtained from the Saudi Ministry of Education & Training. The required sample size was calculated as 378, with probability of achieving statistical significance at 5 % and confidence level of 95%. A total of 388 children [184 (47%) boys and 204 (53%) girls; - age range 3-5 years with a mean of 4.5 (±0.65) were clinically examined. The children were randomly selected from 10 different kindergartens (two public and eight private) children were random selected by stratified method. Approximately, 80% of the preschool children in Riyadh are studying in private kindergartens. These 10 kindergartens were also randomly selected by geographic regions within Riyadh city; two kindergartens each from; Central, Eastern, Western, Southern and Northern Regions.

Ethical approval was obtained from the College of Dentistry Research Center at King Saud University prior to commencement of the study. In addition, approval for this study was obtained from the Ministry of Education & Training. A consent form was used to obtain permission from the parents of the selected children to participate in the study; the form also contained an explanation of the study objectives to the parents.

The intra-examiner reproducibility was assessed on a group of 10 children aged between 3 to 5 years;- that gave a weighted Kappa statistics value of 0.97. The inter-examiner reproducibility (with a senior pediatric dentist) involved a different group of 20 children of similar age group; and gave a weighted Kappa statistics value of 0.93.

The clinical examination was carried out in the children’s schools (after receiving signed consents from the parents) using disposable examination kits and portable light, while child sitting on a portable chair. One examiner carried out all the examinations utilizing WHO criteria (WHO 1997)^19^ for the diagnosis of dental caries (Table-I).

**Table-I:** Modified World Health Organization criteria for diagnosis of dental caries.^19^

Primary Teeth	Status
A	= Sound
B	= Decayed
C	= Filled, and decayed
D	= Filled, no decay
E	= Missing [due to caries] = Missing [others]
F	= Fissure Sealant
G	= Un-erupted tooth

The questionnaire was pre-tested in children and parents not participating in the main study and then appropriate modifications were made to make it more comprehensible for the parents. The examiner visited the selected kindergartens two weeks before the dental examination to distribute the consent forms and questionnaires. The parents completed the questionnaires at home. The questionnaire sought general information on the child’s age and gender as well as demographic information such as parental occupation, education and family size.

All data were entered into a computer using Statistical Program for Social Sciences (SPSS Version 16). Various frequencies were generated. Chi-square test and Z-test were used for comparison between gender, age and children with and without caries. Significance level was p <0.05.

## RESULTS

**Fig.1:**
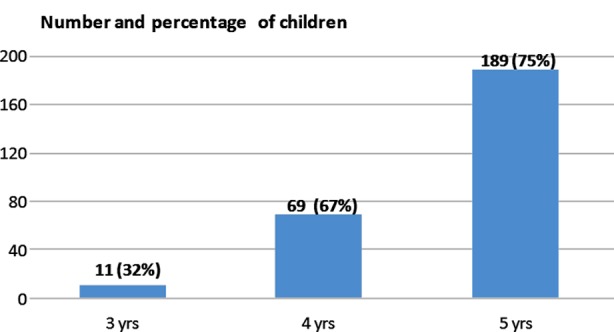
Dental caries prevalence in relation to age. P= 0.229 Not -Sig

Among the total of 388 children examined, 269 (69%) had dental caries with a mean dmft score of 3.4 (3.6±) and dmfs score of 6.9 (9.9±). There was no statistically significant difference (*P =0.592*) between boys and girls in caries prevalence (Table-II). The caries prevalence was high in older children than younger children (32% in 3-year-olds, 67% in 4-year-olds and 75% in5-year-olds children), however, the difference was not statistically significant (*P=0.229*) [Fig. 1].

**Table-II:** The prevalence of dental caries in terms of gender.

Gender	Total	Children with Caries		Children without Caries		P- value

		Number	%	Number	%	
Boys	184	130	71	54	29	*= 0.592*
Girls	204	139	68	65	32	

Total	388	269	69	119	31	--

There was a significant (*P= 0.030*) association between caries prevalence and parental educational level (Table-III). Children of mothers with doctorate/master degree had caries prevalence of 57% while children of mothers with bachelor degree and high school level or below had caries prevalence of 66% and 78% respectively. The children of non-working parents had higher caries prevalence compared to those with working parents (Table-III).

**Table-III:** Dental caries in relation to fathers’/mothers’ education level and work status.

Education	Total	Children with Caries		Children without Caries		P-value

		Number	%	Number	%	
*Doctorate/Master*						Father: 0.366
(Father) (Mother)	69	43	62	26	38	Mother: 0.030
(Mother)	28	16	57	12	43	
*Bachelor*						Father: 0.683 Mother: 0.001
(Father)	187	131	70	56	30	
(Mother)	226	149	66	77	34	
*High school & less*						
(Father)	132	95	72	37	28	
(Mother)	134	104	78	30	22	
*Job*						
Working						
(Father)	366	252	69	114	31	
(Mother)	212	132	62	80	38	
*Non-working/Retired*						
(Father)	22	17	77	5	23	
(Mother)	176	137	78	39	22	

The children from larger family size had higher caries prevalence as compared to those from smaller family size (*P=0.016*) [Table-IV]. There was no significant (*P=0.460*) difference in caries prevalence in relation to birth order of the children.

**Table-IV:** Dental caries in relation to family size and birth-order of the child.

Family size	Total	Children with Caries		Children without Caries		P-value

		Number	%	Number	%	
Two children	64	38	59	26	41	0.016
Three to five children	291	202	69	89	31	
More than five children	33	29	88	4	12	
Order of the child						
First child	119	78	66	41	34	0.460
Middle child	147	102	69	45	31	
Last child	122	89	73	33	27	

## DISCUSSION

The present study has provided important information about prevalence and severity of dental caries among preschool children in Riyadh. The results will be provided to concerned authorities for utilization in designing of future preventive and restorative services.

Al-Agili^20^ conducted a systematic review of childhood caries studies in Saudi Arabia and reported that the National average prevalence of dental caries among Saudi children was approximately 80% in primary dentition. The present study though showed a high (69%) caries prevalence in the studied preschool children, yet it was lower than the National average. A recent study in Jeddah preschool children showed a high prevalence of 89% in the studied sample.^6^ Also, comparing the present study (prevalence 69% and dmft 3.4) with the study by Wyne in 2008^7^ (prevalence 74.8% and dmft 6.1); a clear improvement could be seen especially in severity of dental caries in Riyadh preschoolers. However, caries prevalence of 69% and dmft of 3.4 is still considered seriously high.

Internationally; in United Kingdom, successive National child dental health surveys have shown an improvement in caries prevalence in three-year-old children over four years period starting from 2006 to 2010.^21^ The percentage of children with decay experience was 26% in 2006/7, 25% (2007/2008), decreasing to 18% (2007/2008) and 17% in (2009/2010).^21^

The dmfs score was calculated to determine the severity and treatment needs. The dmfs score in the present study was less than that reported by a study by Al-Malik et al. in, in Jeddah preschool^22^, indicating an improvement in caries severity in preschoolers.

A number of research reports over the past decades have shown that dental caries are linked to social factors.^9^, ^23^ Wigen & Wang^24^ summarized knowledge from the literature regarding parental influence on caries development in preschool children. They concluded that the literature establishes associations between parental factors that are known during pregnancy and early parenthood and caries development in early childhood. The present study showed an association between parent’s educational level and prevalence of caries. The children of highly educated parents had lower caries prevalence. The mother’s educational level appeared to have higher association with caries prevalence in their children than the father’s educational level. Several researchers have found that mothers with higher educational qualifications have children with better dental health.^10^, ^15^, ^16^, ^25^ Hallet & O”Rourke^26^ also showed high incidence of caries in children of mothers with low educational level. These results can be attributed to improved awareness of health related issues and better dental health practices in children of highly educated mothers.

As small families associated with good oral hygiene of their children, large family size was found in one study to have a positive correlation with dental caries.^17^ Abdallah et al.^19^ 2015 investigated the relation between dental caries and socio-demographic factors among Saudi preschool children in Jeddah area, Saudi Arabia. The authors concluded that lower level of parent education, parents Income, socioeconomic status, increased number of children in the family, effect of gender and increased age could be risk factors for increased caries levels. The children from larger families had higher caries prevalence. The association of social factors and dental caries has also been reported by previous studies in other countries.^9^, ^17^ However, in contrast to the other studies,^17^, ^27^ the present study showed no significant difference in caries prevalence between first, middle or last-born child; this finding is in agreement with Wan Salina et al.^28^, while their study reported that birth order was not a significant factor associated with caries experience.

Based on the findings of the present study, preventive educational programs (oral hygiene practices and dietary advice) should be reinforced for the parents (especially mothers) of the preschool children. Pit and fissure sealants need to be placed as early as possible especially on the caries susceptible teeth. Topical fluoride application and fluoride supplements (where indicated) should be prescribed to children with high caries risk.

## CONCLUSIONS

Dental caries prevalence among preschool children in Riyadh, Saudi Arabia is still high. There is a strong negative association between parental education level and caries prevalence in the preschool children. Caries prevalence is high among preschoolers from large families as compared to smaller families. There is no association between caries prevalence and birth-order of the children.
